# Editorial: Anaerobic Energy/Work Supply in Endurance Activities—The Importance and Effect of Computational Method

**DOI:** 10.3389/fspor.2021.777419

**Published:** 2021-11-12

**Authors:** Erik P. Andersson, Dionne A. Noordhof, Jos J. de Koning, Thomas L. Stöggl, Glenn Björklund

**Affiliations:** ^1^Swedish Winter Sports Research Centre, Department of Health Sciences, Mid Sweden University, Östersund, Sweden; ^2^School of Sport Sciences, Faculty of Health Sciences, UiT The Arctic University of Norway, Tromsø, Norway; ^3^Centre for Elite Sports Research, Department of Neuromedicine and Movement Science, Norwegian University of Science and Technology, Trondheim, Norway; ^4^Department of Human Movement Sciences, Vrije Universiteit, Amsterdam Movement Sciences, Amsterdam, Netherlands; ^5^Department of Sport and Exercise Science, University of Salzburg, Salzburg, Austria; ^6^Red Bull Athlete Performance Center, Salzburg, Austria

**Keywords:** critical power/speed, cycling, gross efficiency, maximal accumulated oxygen deficit method, performance testing, time trial, running, sports performance

Physiologically, performance in endurance events is usually described by the total energy provision, i.e., the sum of aerobic and anaerobic metabolic rates, and the gross efficiency (GE) or the gross energy cost (GEC) of movement. Due to the limited capacity of the anaerobic energy supply, the relative anaerobic contribution decreases with exercise duration. Nevertheless, anaerobic energy provision is critical for breakaways and final end-spurts during prolonged endurance events as well as for optimizing pacing strategies over undulating terrain, which emphasizes its importance for endurance performance. While aerobic energy provision during exercise can be quantified by using respiratory measures of oxygen consumption and carbon dioxide production, quantification of anaerobic energy provision is more complicated, and several different methods have been used. The most common approaches are the maximal accumulated oxygen deficit method (MAOD), the GE method, and the critical power (CP) (or critical speed) concept. The current Research Topic aimed to enhance knowledge about anaerobic energy/work supply during endurance activities, with a specific focus on methodological issues. In all, six original articles were accepted.

Andersson et al. compared four different models for estimating anaerobic energy supply during treadmill running. Two linear speed-metabolic rate regression models (based on five submaximal stages) were used to estimate the required metabolic rate during a 4-min time trial (TT), either including (5+Y_LIN_) or excluding (5-Y_LIN_) a measured y-intercept. Also, the average GEC (GEC_AVG_) based on all submaximal stages, or the GEC based on the last submaximal stage (GEC_LAST_), were used to estimate the required metabolic rate during the TT. The findings were that GEC was speed independent, on a group level, and that 5-Y_LIN_, GEC_AVG_, and GEC_LAST_ generated similar anaerobic capacities, while the 5+Y_LIN_ model generated a 26% lower value of anaerobic capacity due to the significantly lower regression slope. Based on the results, the 5-Y_LIN_ model was suggested to be the most reliable and valid model.

Noordhof et al. analyzed the dynamics of the anaerobic energy contribution during high-intensity skate roller-skiing on a simulated course and compared three different models of estimating anaerobic energy/work contribution. The models used were two linear regression models and a GE model. All three models generated similar average values of anaerobic work but with a high individual variation, which suggests that the different methods should not be used interchangeably. During the ~13-min high-intensity effort, the relative energy contributions were 88% aerobic and 12% anaerobic.

To predict endurance performance based on physiological and external factors, as well as to improve pacing strategies through numerical simulations, a valid and reliable bioenergetic model can be a valuable tool. In this context, Lidar et al. developed a method for individual parameter estimation of four bioenergetic models and assessed the validity and reliability of these models in their continuous prediction of aerobic and anaerobic metabolic energy utilization during two successive roller-skiing sprint time trials (STTs). Aerobic and anaerobic metabolic rates, external power output, and GE were measured. In general, all four bioenergetic models generated valid and reliable results during the first STT but were less reliable and valid during the second STT.

The transition from heavy- to severe-intensity exercise in endurance sports defines the change from mainly aerobic energy contribution to a distinctive increase in anaerobic energy contribution. Traditionally, this change in exercise intensity has been defined using various types of lactate threshold concepts. A concept that has been used to define the transition from heavy- to severe-intensity exercise in a similar way as lactate thresholds is the CP concept. In this context, Valenzuela et al. have presented how various lactate thresholds relate to CP in cycling. Although associations between lactate thresholds and CP were found, a major concern is the wide range of limits of agreement between methods, which suggests that different methods should not be used interchangeably.

In many endurance sports, the exercise intensity and energy contributions are highly variable and depend on race tactics as well as the course profile. One potentially disregarded factor is the importance of an intermittent anaerobic energy contribution for endurance performance. In the study by Næss et al. power output data from a mountain bike (MTB) race gave insight into the extent of variability in exercise intensity. Even though the average power output remained below CP, a considerable time (~40%) was spent in the severe exercise-intensity domain, taxing the anaerobic energy system. Additionally, the significant depletion of the anaerobic work capacity (*W*') during the latter stages of the race shows a considerable need for anaerobic energy/work supply, which highlights the importance of anaerobic work capacity as a factor for MTB performance.

Almquist et al. provided new and important insights for cyclists on how to improve anaerobic power and capacity. The study was conducted in a controlled laboratory setting and provided evidence that the decline in power output during repeated sprints was primarily caused by a decrease in anaerobically attributable power, with a resultant increase in relative aerobic power contribution. Furthermore, the study included a training intervention with the inclusion of sprints during low-intensity training for 2 weeks. Interestingly, the intervention group that was exposed to sprints during low-intensity training, increased their repeated sprint ability compared to the control group. These results suggest that with a relatively small effort cyclists can improve their anaerobic capacity and power within a short time frame.

These six Research Topic articles cover some of the methodological issues related to the complex nature of estimating anaerobic energy/work supply during running, roller-skiing, and cycling exercise. However, due to the complex nature of quantifying anaerobic energy supply and the different methods that have been used in previous research, there is still a relatively sparse number of published studies with a specific methodological purpose. For example, the agreement between *W'*, calculated based on CP, and anaerobic work capacity based on the GE and/or MAOD method(s) has been sparsely studied.

## Author Contributions

EA and GB wrote the first draft of this editorial. All authors drafted the editorial and approved the final version.

## Funding

This work was supported by the Swedish National Centre for Research in Sports (CIF, P2020-0157).

## Conflict of Interest

The authors declare that the research was conducted in the absence of any commercial or financial relationships that could be construed as a potential conflict of interest.

## Publisher's Note

All claims expressed in this article are solely those of the authors and do not necessarily represent those of their affiliated organizations, or those of the publisher, the editors and the reviewers. Any product that may be evaluated in this article, or claim that may be made by its manufacturer, is not guaranteed or endorsed by the publisher.

